# Targeted Germline Sequencing Identifies Ultra-Rare DDR Variants in High-Risk Prostate Cancer: Short title: Germline DDR Variants in High-Risk Prostate Cancer

**DOI:** 10.31661/gmj.v15i.4248

**Published:** 2026-08-10

**Authors:** Rawaz Rizgar Hassan, Abdulkarim Yasin Karim

**Affiliations:** ^1^ Department of Biology, College of Science, Salahaddin University-Erbil, Kirkuk Road, 44001 Erbil, Kurdistan Region, Iraq

**Keywords:** Prostate Cancer, Whole Exome Sequencing, Germline Mutation, DNA Damage Repair, Precision Oncology

## Abstract

**Background:**

Prostate cancer (PCa) is a biologically heterogeneous disease, with a subset of cases driven by hereditary germline alterations.

**Methods:**

Targeted germline sequencing using the Twist Fixed Panel was performed in nine high-risk PCa patients (median age 47; range 30–61). Variants were classified based on ClinVar, gnomAD, and in silico prediction tools.

**Results:**

Pathogenic and likely pathogenic variants in DNA damage repair (DDR) genes were identified, including BRCA2, BRIP1, PALB2, and BRCA1. A homozygous ATM variant was observed in one patient. Several ultra-rare variants in MLH3 and BARD1 were also detected. Most variants had allele frequencies <10-6.

**Conclusions:**

High-risk and early-onset PCa is associated with rare germline DDR alterations. These findings support expanded germline testing in younger patients and suggest potential relevance for precision oncology approaches, although functional validation is required..

## Introduction

Prostate cancer (PCa) remains a significant global health challenge, characterized by profound biological heterogeneity and variable clinical trajectories. Although a large proportion of cases are indolent, a key subset of patients presents with an aggressive phenotype including rapid disease progression, treatment resistance to standard treatments and high mortality. Recent advances in molecular stratification have improved understanding of this heterogeneity, highlighting changes such as PTEN loss, TP53 amplification, and TMPRSS2-ERG fusions which establish individual aggressive subtypes [1, 2]. Nevertheless, for those patients with early onset and high risk the clinical burden may be due to a central hereditary factor which is not adequately reflected by classical markers. Germline mutations in DNA damage repair (DDR) pathways, which occur in roughly 10–16% of men with metastatic or high-risk PCa [3, 4], strongly mold the genomic architecture of high-risk PCa.

Mutations in the Homologous Recombination (HR) pathway, especially BRCA2, that are the most frequent and are consistently linked to adverse outcomes, such as shorter cause-specific survival in metastatic castration resistant prostate cancer (mCRPC) [5]. However, beyond BRCA2, other DDR genes including ATM, CHEK2, and PALB2 have emerged as clinically relevant genes that not only dictate prognosis but affect therapeutic sensitivity to Poly (ADP-ribose) Polymerase (PARP) inhibitors and second-generation hormonal agents like abiraterone [6, 7].

Despite the established role of these high-penetrance genes, a significant gap remains in understanding the germline landscape of early-onset PCa. Traditional screening and genome-wide association studies (GWAS) have largely focused on common polymorphisms, which often possess small effect sizes. In contrast, emerging research indicates that ultra-rare germline variants—specifically “private” protein-coding and structural variants-contribute significantly to cancer susceptibility with substantially stronger effect sizes than common Single Nucleotide Polymorphisms (SNPs) [8, 9]. In early-onset cohorts, these rare variants may explain a measurable fraction of cancer liability that is often missed when using standard age cutoffs or limited genetic panels [10]. Identifying these rare hits is essential, as they often have more direct and substantial impacts on cancer predisposition and subsequent tumor evolution [11].

As the field shifts toward Precision oncology, there is a critical need to differentiate these pathogenic variants from the background of common genetic variation. This study aims to investigate the germline architecture of an early-onset, high-risk prostate cancer cohort using next-generation sequencing. By characterizing pathogenic and ultra-rare variants across HR and Mismatch Repair (MMR) pathways, we seek to interpret how these specific genetic hits dictate clinical presentation and provide a robust framework.

## Materials and Methods

### Patient Selection and Ethical Considerations

The study cohort comprised high-risk male patients diagnosed with PCa. Selection criteria included early-onset disease (age ≤60 years), a high clinical risk profile, and/or a positive family history of malignancy. Informed consent was obtained from all participants prior to sample collection.

### Genomic DNA Extraction and Quality Control

Peripheral blood was collected in EDTA tubes. Genomic DNA (gDNA) was extracted from whole blood using the PureLink™ Genomic DNA Mini Kit (Thermo Fisher Scientific, USA) following the manufacturer’s instructions. Briefly, samples underwent enzymatic lysis with Proteinase K and RNase A at 55°C for 10 minutes, followed by ethanol precipitation and column-based purification. The purified gDNA was eluted in 25–200 μL of Elution Buffer. Quantification and quality assessment were performed using the OneDrop TOUCH NanoDrop (Biometrics Company); an A260/280 absorbance ratio of ~1.7–1.8 was considered acceptable for DNA purity.

### Library Preparation and Enzymatic Fragmentation

Targeted library construction was performed using the TWIST Library Preparation EF 2.0 with Enzymatic Fragmentation kit (Twist Bioscience, USA). For each sample, 25 ng of input gDNA was diluted to a 20 μL volume. Enzymatic fragmentation, end-repair, and dA-tailing were conducted in a thermal cycler (Applied Biosystems™ SimpliAmp™) with a specific profile: 4°C (Hold), 37°C (11 min), and 65°C (30 min). Subsequently, MGI-specific universal adapters were ligated to the dA-tailed fragments at 20°C for 15 minutes. The adapter-ligated libraries were purified using DNA Purification Beads (Twist Bioscience) and amplified via PCR (9 cycles) using Twist UDI Primers to generate a sufficient DNA mass for capture. Following PCR, libraries were quantified using the Qubit 4 Fluorometer (Thermo Fisher Scientific). Target enrichment was executed using the Twist Target Enrichment Standard Hybridization v1 Protocol. Pooled indexed libraries (5–10 μL) were hybridized with a custom probe panel (Twist Fixed Panel) for 16 hours at 70°C. Post-hybridization capture was performed using streptavidin-coated magnetic beads, followed by stringent washing at 65°C and final elution.

DNA Nanoball (DNB) Generation and Sequencing The enriched libraries were prepared for sequencing according to the MGI Protocol. The double-stranded libraries were heat-denatured and circularized into single-stranded DNA (ssDNA) using the MGIEasy Circularization Kit (MGI, China). Linear DNA remnants were removed via enzymatic digestion. The ssDNA circles were subsequently amplified via Rolling Circle Amplification (RCA) to form dense DNA Nanoballs (DNBs). Quantification of ssDNA and DNBs was performed using the Qubit ssDNA Assay Kit. High-throughput sequencing was conducted on the DNBSEQ-G400 platform (MGI, China).

### Bioinformatics Pipeline and Variant Interpretation

No inferential statistical analyses were conducted; results are descriptive only. Raw sequencing data were processed using the Genomize-Seq bioinformatics platform (version 3.0.5). Alignment, variant calling, and annotation were performed against the GRCh38 human reference genome. Variants were filtered and prioritized based on:

 1. Clinical Significance: Cross-referencing with ClinVar (v2024).

 2. Population Frequency: Comparison with gnomAD v4.1.0; variants with an Allele Frequency (AF)>0.01 in the general population were excluded.

 3. In Silico Prediction: Functional impact assessment using SIFT (deleterious threshold <0.05) and PolyPhen-2 (damaging threshold >0.85).

4. Zygosity: Classification into Heterozygous (HET) or Homozygous (HOM) based on Variant Allele Frequency (VAF). Ethical Approval Ethical approval for this study was obtained from the Human Research Ethics Committee (HREC), College of Science, Salahaddin University-Erbil, Kurdistan Region, Iraq. The approval was issued for the project entitled “A Multi-omic Exploration of Prostate Cancer Biomarkers through MicroRNA Expression Profiling, Gene Expression, and Whole Genome Sequencing” under Reference No.: 45/44, dated 12/03/2025. All participants provided informed consent before sample collection, and the study was conducted in accordance with the ethical principles of the Declaration of Helsinki.

## Results

**Table:** Table1. Molecular and population genetic characteristics of identified germline variants

ID	Age	Gene	Variant (Protein)	ClinVar	Zygosity	gnomAD AF
4	30	BRCA2	p.Arg8AlafsTer5	Pathogenic	HET	6.19×10−7
1	34	BRIP1	p.Arg439Ter	Pathogenic	HET	3.72×10−6
2	38	BARD1	p.Ile169Val	VUS	HET	6.20×10−7
9	43	PALB2	p.Lys569ArgfsTer29	Pathogenic	HET	6.20×10−7
3	47	ATM	p.Val2716Ala	VUS/LP	HOM	2.73×10−5
8	51	BRCA1	p.Gln1612Ter	Pathogenic	HET	<10−5
6	61	MLH3	p.Gly1319Glu	VUS	HET	<10−5

HET: heterozygous; HOM: homozygous; AF: allele frequency; VUS: variant of uncertain significance; LP: likely pathogenic; DDR: DNA damage repair.

### Patient Cohort and Clinical Characteristics

Targeted germline sequencing was performed on a cohort of high-risk PCa patients characterized by early disease onset and significant clinical indicators of aggressiveness. The cohort (N=9) exhibited a median age at diagnosis of 47 years (range: 30–61 years). All patients were classified as “High Risk” based on clinical criteria, with 88% (8/9) of the cohort possessing a positive family history of malignancy. The sequencing analysis yielded a mean coverage depth of 124×, with a minimum coverage depth of 52×. At the relevant threshold, 90% of target bases were successfully covered. These values reflect the descriptive characteristics of the dataset and summarize the overall sequencing performance without inferential statistical testing. Read quality thresholds and variant calling filters were not manually configured in this study. The software employed integrates these parameters automatically within its pipeline, and therefore they are not directly accessible for adjustment or reporting. As a result, quality control and variant filtering were applied internally by the system rather than through user-defined settings.

### Genomic Landscape of Pathogenic DDR Variants

Comprehensive germline analysis identified pathogenic or likely pathogenic variants in high-penetrance DNA Damage Repair (DDR) genes across multiple patients. A frameshift deletion in BRCA2 (p.Arg8AlafsTer5) was identified in the youngest patient of the cohort (Patient #4, age 30). In another early-onset case (Patient #9, age 43), a truncating deletion in PALB2 (p.Lys569ArgfsTer29) was detected. Nonsense Mutations (Stop-Gain): High-impact stop-gain mutations were identified in BRIP1 (p.Arg439Ter) in a 34-year-old and BRCA1 (p.Gln1612Ter) in a 51-year-old. These variants are predicted to cause premature truncation of the helicase and C-terminal domains, respectively.

### Homozygous ATM Finding

A notable genomic finding was observed in patient #3 (age 47), who was found to be homozygous for the ATM (p.Val2716Ala) missense variant. Variant Allele Frequency (VAF) analysis confirmed 100% occupancy at this locus. This homozygous “double-hit” genotype is exceptionally rare; while heterozygous carriers of this variant are present in global datasets, there no homozygous individuals have been reported among the 1,613,980 alleles sequenced in gnomAD v4.1.0.

Mismatch Repair (MMR) and BARD1 VUS Analysis Beyond the classical HRR pathway, an MLH3 (p.Gly1319Glu) variant was identified in patient #6 (age 61). While currently classified as a Variant of Uncertain Significance (VUS), in silico predictive algorithms suggested a deleterious impact, with a SIFT score of 0.01 and a PolyPhen-2 score of 0.998. Additionally, an ultra-rare BARD1 (p.Ile169Val) variant was identified in patient #2 (age 38).

### Population Rarity and Database Analysis

Comparison with the gnomAD v4.1.0 population database revealed that the majority of identified variants are categorized as ultra-rare, defined by an Allele Frequency (AF) <10−6. (Table1)

## Discussion

The identification of pathogenic germline variants in nearly all patients within this high-risk cohort reinforces the growing consensus that early-onset and aggressive PCa are driven by a distinct genetic architecture. By shifting the focus from common polymorphisms to ultra-rare genomic variants, our findings may provide an important insight into biological interactions spanning the Homologous Recombination (HR) and Mismatch Repair (MMR) pathways that dictate the clinical trajectory of this disease.

The most striking observation in our cohort was the diagnosis of a 30-year-old patient harboring an ultra-rare BRCA2 frameshift mutation (p.Arg8AlafsTer5).

Current literature characterizes BRCA2 as a high-penetrance driver that confers a 4.45-fold increased risk of PCa and is associated with distinct evolutionary trajectories leading to lethal phenotypes [12, 13]. The detection of such a high-impact mutation in a 30-year-old underscores that severe genomic instability can compress the traditional timeline of oncogenesis by decades. This is further supported by our finding of a BRIP1 (p.Arg439Ter) nonsense mutation in a 34-year-old. While BRIP1 is less frequent than BRCA2, truncating mutations in this gene are specifically linked to young-onset susceptibility [14]. Both BRCA2 and BRIP1 carriers represent a patient with germline DDR mutations for Precision oncology; the deficiency in DNA repair makes these tumors uniquely sensitive to PARP inhibitors like Olaparib, which exploit the principle of synthetic lethality [15, 16].

In the ATM gene, a homozygous variant was identified (p.Val2716Ala) in a 47-year-old patient. While heterozygous ATM mutations increase PCa risk fourfold and are markers of lethal disease [17, 18], the clinical implications of homozygosity are rarely documented in adult oncology. The complete absence of homozygous carriers for this variant in large-scale population databases suggests that this genotype represents a potential biological susceptibility. Therapeutically, the absolute loss of ATM defines a specific subtype that may be consistently sensitive to ATR inhibitors and radiotherapy [19].

It is important to understand the functional roles of BRCA1 and BARD1 variants in their target (androgen receptor) signaling schema. AR signaling affects BRCA1 protein expression, as both a marker of tumor progression and a modulator of antioxidant defense [20]. In this 51-year-old patient, the germline loss of BRCA1 (p.Gln1612Ter) most likely limits these defenses and results in the more aggressive aneuploid phenotypes observed in carriers younger than 65 [12]. At the same time, that too, the ultra-rare BARD1 variant that has recently been diagnosed in a 38-year-old has provided a more nuanced view of the risk. While BARD1 variants do not confer widespread risk for PCa in the average male 22, its important role in the formation of a functional E3 ubiquitin ligase complex with BRCA1 makes BARD1 loss an important marker for homologous recombination deficiency (HRD). So “secondary” HR genes presumably act on these early-onset tumours due to synergistic repair failure that sensitizes tumours to such PARP inhibitors [18].

In our cohort we included an MLH3 variant that brings the Mismatch Repair (MMR) pathway to the clinical dialogue. Despite the rarity of MMR alterations, it is critical to recognize these alterations which are associated with poor abiraterone response and shorter progression-free survival [20]. This finding provides an immediate clinical directive: if patients have MLH3 or other MMR deficiencies, clinicians should consider taxane chemotherapy or immunotherapy rather than second-generation hormonal therapies, as the underlying genomic instability facilitates a rapid escape from hormonal control.

We find one recurring thread in this study: the very rareness of the variants identified. Population data (gnomAD) suggest that most variants in our patients occur in fewer than one in a million people worldwide. From the perspective of early-onset disease, this “ultra-rarity” provides strong circumstantial evidence of pathogenicity. High-penetrance alleles that cause disease at age 30 or 40 are under great negative selective pressure and are thus unlikely to be common in the general population. This may support the classification of several “Variants of Uncertain Significance” (VUS) as likely contributors to the disease phenotype based on their rarity and high in silico deleterious scores.

Our data align with the results of the IMPACT study, which advocates for systematic PSA screening in known carriers of DDR mutations [20]. The presence of these variants in patients as young as 30 and 34 suggests that current screening guidelines, which typically begin at age 50, are insufficient for hereditary cohorts. We suggest for a lower threshold for germline testing in any man presenting with high-risk clinical features or early-onset disease. Identifying these carriers may help to allow for a paradigm shift from reactive treatment to proactive precision medicine, utilizing specific vulnerabilities like PARP inhibition and ATR sensitization while enabling life-saving cascade testing for their families.

A primary limitation of this study is the relatively small sample size, as a total of nine samples were evaluated. Next-Generation Sequencing (NGS) remains a high-cost methodology, which inherently constrained the scale of sample collection and sequencing depth for this cohort. Because of these financial and logistical boundaries, this work should be interpreted as an exploratory study, designed to identify initial trends and generate hypotheses rather than provide definitive epidemiological or statistical generalizations. Furthermore, a traditional experimental control group was not processed alongside the samples. Instead, to establish a baseline for comparison, data were benchmarked against established reference sequences available in curated bioinformatics databases. While database comparisons are a widely accepted approach in exploratory genomics, future large-scale studies with concurrent control groups are necessary to validate these preliminary findings and fully characterize the observed genetic variations.

## Conclusion

This study demonstrates that early-onset and high-risk prostate cancer is associated with a significant burden of rare and ultra-rare germline variants in key DNA damage repair (DDR) genes, particularly BRCA2, BRCA1, PALB2, BRIP1, and ATM. The presence of high-impact and extremely low-frequency variants supports a role for inherited DDR deficiency in aggressive disease development.

These findings highlight the clinical importance of germline testing in younger and high-risk patients, with direct implications for precision oncology approaches such as PARP inhibition and family risk assessment.

However, the small sample size and exploratory nature of the study require caution, and functional validation in larger cohorts is needed to confirm pathogenicity.

Overall, this work supports the integration of expanded germline sequencing into prostate cancer evaluation to improve risk stratification and guide targeted treatment strategies.

## Conflict of Interest

The authors declare that there are no conflicts of interest regarding the publication of this study.

## AI Disclosure Statement

During the preparation of this work, the authors used ChatGPT for language editing and grammatical correction. After using this tool, the authors reviewed and edited the content as needed and take full responsibility for the content of the published article.
